# Examining the green factors affecting environmental performance in small and medium–sized enterprises: A mediating essence of green creativity

**DOI:** 10.3389/fpsyg.2022.1078203

**Published:** 2022-12-14

**Authors:** Di He, Ali Raza, Min Chen, Yiwei Xu, Otsile Morake

**Affiliations:** ^1^School of Management, Jiangsu University, Zhenjiang, China; ^2^Department of Business Administration, Sukkur IBA University, Sukkur, Sindh, Pakistan; ^3^School of Management, Zhejiang Gongshang University, Hangzhou, China

**Keywords:** green transformational leadership, green activities, green knowledge, green creativity, environmental performance

## Abstract

Despite a large amount of literature on the management and sustainability of green enterprises, representatives’ contributions to environmental challenges have received scant attention. This study purposefully assesses how managers’ ecological expertise and ability to transform organizations’ leadership practices into more environmentally friendly ones, with the help of green creativity (GC) as a mediating factor. The study utilizes partial least square structural equation modeling to examine the perceptions of 400 respondents in various leadership roles in the small and medium businesses industry. The study’s findings point to the beneficial impacts of green knowledge (GK), green transformational leadership (GTL), and GC on environmental performance (EP). GC also appears to perform a meaningful mediating role in the links between GK and EP, GTL, and EP. The primary takeaway from recent research is that participants in the sector may be able to respond with green efforts that are specific to their businesses with the support of managers’ environmental concerns. There is a discussion on practice recommendations and future directions.

## Introduction

It has become increasingly evident that academics, stakeholders, and policymakers are urging companies to adopt sustainable strategies based on environmental performance (EP) (Roscoe et al., 2019; Li and Ramanathan, 2020). Environmental awareness and green marketing are capturing constructive acceptance and attention globally (Obeidat et al., 2020). The goal of companies is to encourage sustainable business activities and create innovative brands that shield the environment at the same time. In order to improve EP, proactive attitudes to green creativities are implanted in leadership, knowledge, and organizational skills (Song et al., 2020). A company’s EP is a primary target when seeking to increase efficiency and competitiveness and reduce costs (Daddi et al., 2019; Amoako et al., 2020).

In response to (SDGs) sustainable development goals and a greener agenda, industry attention has shifted to green marketing, green HRM, green innovation, and green supply chain. In addition, organizational goals are influenced by institutional factors like human capital, business, and commercial components, infrastructure, technology, creativity, and knowledge (Shahzad et al., 2020). Green transformational leadership (GTL) performs a vibrant role in improving the EP of any venture. Studies have examined the relationship between GTL and environmental management systems and EP in companies (Li et al., 2020; Pham et al., 2020; Singh et al., 2020). It is suggested by these researchers that GTL affects EP indirectly by a variety of factors (Singh et al., 2020) and other green human resource management actions (Roscoe et al., 2019). EP and GTL are not directly linked. However, Li et al. (2020) discussed how GTL affected employee motivation, which indirectly affected an organization’s energy-boosting and reconditioning capacity.

To solve environmental problems, organizational capacity, such as green knowledge (GK), is considered a strategic resource (Seman et al., 2019). Environmental or GK has been promoted in many emerging countries through education programs addressing environmental issues. As part of government and entrepreneurial activities, knowledge, skills, and values respecting the environment are also emphasized(Mohiuddin et al., 2018). It is still unclear how managers’ GK affects EP. While the relationship between environmental management and green creativity is deep-rooted (Song et al., 2020), as well as the function of green creativity (GC) as a mediator (Zameer et al., 2020), we discuss that any research on GTL should take a closer look at their GC. The ability to be creative is broadly acknowledged as a factor in competitive advantage, superior organizational performance, and resolving customers’ problems (Darvishmotevali et al., 2020).

The literature also discusses creativity as a factor in environmental management systems. In terms of GC study, there is still a lack of academic research (Cheng, 2019). Several researchers believe that green creativity is crucial in connecting various green human resource management practices to environmental management systems. A critical justification for ecological management system research is the intermediating role of GC (Tuan, 2020). In the same way, stakeholders, governments, and customers are concerned about the enterprise’s sustainability.

Anwar and Li (2021) stated that it is encouraged to SMEs to work for environmental issues and stand for societal value. Besides, several forms of Pakistani SMEs i.e., manufacturing, trading, and services (Fang et al., 2022) education, healthcare (Qalati et al., 2022a) and other services (Qalati et al., 2022c). In addition, numerous natures are available (SMEDA, 2020) participate at economic performance and majority remain fruitful for environmental productivity.

Businesses may employ green innovation to distinguish themselves from existing opponents and can also be employed to highlight environmental needs in the commercial sector (Ding et al., 2022). In addition, it has been argued that additional research is required to comprehend managers’ role in maintaining environmental sustainability (Pham et al., 2020). Researchers recommend studying transformational leadership to achieve particular environmental goals (Li et al., 2022). Our hypothesis, therefore, is that enterprises’ EP may be linked to GTL. The concept of GC needs to be given more attention in environmental management. Most environmental management research has engaged green innovation from employees, managers, and organizations (Jia et al., 2018; Song and Yu, 2018). As part of the study, GK, GTL, GC, and EP will be investigated to fill in the gaps identified, so that policy implications for addressing these issues can be developed. We must address environmental challenges as soon as possible (Yusoff et al., 2020). Accordingly, the research probes the relationship between GK, GTL, and enterprise EP, along with the mechanisms through which GC mediates these interactions. The study identifies the indirect mediating channel between GC and EP by creating a hypothetical model to fill in the gaps. To test the concept, information was gathered from the Pakistan enterprise sector. Qalati et al. (2021b) provided the participation of SMEs, including Pakistan, in different services and their contribution is added in economic activities. Four thematic areas of the study are included in the remaining sections of the study, such as the underlying theory and literature review, the formulation of hypotheses, the methodology, the empirical findings, and the interpretation, as well as the discussion, which highlights potential directions for future research as well as limitations in current research.

## Theoretical background and hypotheses development

The research’s theme model is grounded on the resource-based view (RBV), social identity theory, and organizational creativity theory to study the sector’s HR-Creativity-Performance nexus. The idea that company performance depends on human capital is widely acknowledged in literature on human resource management and strategy (Takeuchi et al., 2007). Business performance relies upon the ability to make use of resources that are expensive and hard to replicate by rivals. In the view of RBV, a company’s ability to use strategic resources guarantees sustained performance and boosts competitive benefit through various methods (Barney and Wright, 1998). RBV and human resources are linked to GTL, EP, and GK, which are crucial resources supporting green human resources (Boxall and Steeneveld, 1999).

It is argued that human capital performs a vital role in corporate social systems, but its unique qualities—green expertise and transformational leadership, for instance—can help companies improve their environmental management systems and their EP (Takeuchi et al., 2007). Organizing creativity and social identity theory suggests that people join groups that enhance their sense of self and understanding (Woodman et al., 1993; Hogg, 2001). Identifying intra-individual elements is key to social identity theory and organizational creativity. It tends to be easier for people to identify with groups that take a positive stance and are enthusiastic about developing fresh, original ideas to enhance their self-esteem.

Moreover, optimistic managers about environmental management systems practice environmentally friendly conduct and are open to new concepts that aid the firm in achieving its environmental protection goals (Song and Yu, 2018). Using the RBV, managers were studied as a mean of performance in improving environmental settings. Human resources are one of the mediums of organizational effectiveness (Omondi-Ochieng, 2019). Social identity theory also serves as a theoretical lens through which we evaluate green leadership and green transformative knowledge within the framework. Based on the assessment of self-concept, individuals express themselves in any organization on the basis of their knowledge, values, and social understanding.

Individuals with an innate connection to an organization develop attitudes and behaviors based on their affiliation to a specific group (De Roeck and Maon, 2018). Using organizational creativity theory, we examine GC, which aims to address environmental issues and comply with environmental regulations. The organization is open to new and practical ideas that can improve EP across all spheres. As a result, businesses adopt novel concepts that ultimately enhance resource efficiency and foster GC in the workplace (Song and Yu, 2018).

### Environmental performance and green creativity

Green creativity leads to eco-friendly behavior and improves organizational performance. Creativity enhances the effectiveness of environmental management systems by implementing eco-friendly policies and procedures (Cheng, 2019; Song et al., 2020). According to the practical findings, the team’s creativity is crucial when adopting green marketing strategies. Green marketing tactics and combinations improve performance and give businesses a competitive edge (Jia et al., 2018). The argument is that companies should foster creativity as part of their focus on managing environmental systems and developing novel solutions to any problems that arise (Dangelico et al., 2017). Innovative green practices encourage businesses to make eco-friendly investments, eco-planning decisions, and investment recoveries that benefit both the environment and businesses. EP requires researchers to overlook the essential component of success, which is green innovation, while concentrating on other organizational capabilities. The strategic management of the environment requires organizations to be open to change, to respect the environment when developing new products, processes, and technologies, and to change how existing strategies are implemented (Darvishmotevali et al., 2020). Firms should prioritize creativity in order to oversee innovative performance. To fulfill expectations, social needs, and environmental goals, managers should focus on developing products and processes that augment product performance. Innovative solutions and procedures can make new products/services more profitable (Chen et al., 2016). Creativity cultivates an environment conducive to increasing environmental commitments and reducing environmental damage (Aeknarajindawat and Jermsittiparsert, 2019; Song et al., 2020). We, therefore, proposed the subsequent:

**H1:** There is a strong connection between EP and GC.

### Green knowledge and environmental performance

Employees with GK are better able to understand the ecosystem, show concern about any negative effects their operations may have on the environment, and appreciate the system of shared responsibility for sustainable development (Martinez-Martinez et al., 2019). Law et al. (2017), Mohiuddin et al. (2018) and Wang et al. (2018) argue that green information influences managers’ and customers’ decisions. As green initiatives become more widespread, organizations increasingly recognize “knowledge” as a driving force. The relationship between knowledge and conduct is well supported by prior research (Berchicci et al., 2019). Managers’ expertise is also essential for implementing green marketing and creating environmental initiatives (Chan et al., 2020). Further research domains need a wide and concrete approach to examine businesses’ experience regarding their key stakeholders’ participation in eco-friendly organizational practices (Tang et al., 2018).

In order to improve organizational performance and transform businesses into environmentally sustainable ones, it is essential to understand environmental issues and solutions accurately, thoroughly, and promptly (Mohiuddin et al., 2018; Chan et al., 2020). Environmental expertise is used to formulate internal environmental protection policies. A successful green staff training program depends heavily on the support and expertise of senior management in order to be adopted and implemented successfully for employees (Kim et al., 2019). An environmentally aware management team is crucial to developing, embracing, and implementing green marketing strategies. Further, managers’ ecological expertise helps them approach challenges more creatively and environmentally friendly (Saeed et al., 2019). In the present environmental age, companies require to develop an environmental management attitude to motivate their green innovations (Chen et al., 2014). Thus, we hypothesize as

**H2**: EP is positively correlated with GK.

### Green knowledge and green creativity

Creative thinking is the process of conceiving new and valuable products, ideas, or services. GC is creating new, practical, and beneficial concepts that lead to environmentally sustainable procedures, products, services, and practices (Li et al., 2020). All green activities and initiatives are based on green ingenuity (Chen et al., 2016). Innovation and production of green products all begin with green creativity (Chen et al., 2016; Tuan, 2020). GC is essential in the environmental management system because any novel concept requires it. In an integrated environmental management system, no concept is retained that lacks innovation (Ogbeibu et al., 2020). A key component of the environmental management system is the need to approach routine tasks creatively and innovatively. In this respect, the foundation for any green practice or initiative can be considered green creativity in environmental management systems (Ogbeibu et al., 2020). Some individuals are naturally creative, and others necessitate a learning process to obtain knowledge that will lead to creativity (Cheng, 2019). Numerous studies have examined the relative importance of information for creative thinking. Creative notions may occasionally be made more valuable by the type of knowledge they are based on. There are also a variety of knowledge frameworks available to support the development of creative solutions and original ideas (Cheng, 2019). Albort-Morant et al. (2016) also relate this idea to improving the organization’s green and creative cultures. Organizations adopt green initiatives for various reasons, which can be divided into macro and micro forces. Organizational green culture, managerial characteristics, values, and property status are micro-factors, while regulatory pressure, stakeholder demands, and changes in customer preferences are macro-factors (Jia et al., 2018).

Moreover, management’s awareness and interest in environmental issues influence organizational inventiveness about environmental issues, which leads to proactive environmental practices (Jia et al., 2018; Liao and Chen, 2018). As a result, the managers’ failure to utilize their knowledge will not negatively impact the group’s success. By adding knowledge to intelligent reasoning, any concept may become more distinctive and new (Sung and Choi, 2012; Cheng, 2019). As a result,

**H3**: GC and GK are positively correlated.

### Green transformational leadership and environmental performance

Using the strategic leadership theory, Chen and Chang (2013) assert that environmental and social responsibility success depends on the degree of transformational leadership within the organization. According to Chen et al. (2014), leadership that promotes green transformation involves motivating employees to achieve environmental objectives. A green transformational leader establishes an environment where the organization adheres to high environmental standards. Transformative leaders support their followers while inspiring them to view things differently. In addition, GTL leads to better performance in green product development by articulating a larger perspective of the environment, establishing environmental standards, and motivating followers with environmental standards and goals (Jia et al., 2018). Studies have identified several challenges to adopting and using green marketing methods. In order to implement green practices effectively, intermediate problems like the lack of expert advice, maintenance costs and increasing implementation, and management’s unwillingness to share information about green practices and skills must be highlighted (Singh et al., 2020) must be addressed. Transformational green leaders encourage creative solutions to environmental problems and support corporate innovation. Transformational leadership benefits EP and inventive performance (Yang and Yang, 2019). The environmental sustainability in the service industry is impacted by GTL (Çop et al., 2021). This led to the formulation of a study.

**H4:** There is a positive relation between EP and GTL.

### Green transformational leadership and green creativity

A transformational leader demonstrates innovative and creative conduct, which plays a role model for creativity in the workplace. Innovative ideas and innovative outcomes are fundamentally influenced by transformational leaders (Mittal and Dhar, 2016). In environmental management, the four features of transformational leadership- intellectual stimulation, charisma, individual consideration, and inspiring motivation- substantially impact how people behave within a company (Mi et al., 2019). When transformational leadership stimulates followers’ intellectual processes, they are able to use information research to generate constructive solutions to problems. By considering each member’s specific needs, managers can provide coaching and guidance that fosters the group’s creativity. Transformational leadership creates an environment where a clear vision is given to the team, igniting and fostering a culture of creativity (Mittal and Dhar, 2016). The followers of transformational leaders with an inspiring drive to protect the environment receive adequate inspiration, encouragement, and enthusiasm to support their initiatives (Adıgüzel, 2019), the effectiveness and creativity of the leader (Jia et al., 2018), readiness to meet customer demands and environmental regulations (Liao and Tsai, 2019), and leadership to take in employees and the creation of a green culture are all mentioned in the literature that is currently available (Song and Yu, 2018). In order for an organization to be creative, leaders need to demonstrate traits that encourage creativity within it. Organizations that want to foster creativity should practice transformational leadership. In addition, Jia et al. (2018) found that transformational leadership impacts and signifies employee creativity. Consequently, this research recommends the resulting hypothesis:

**H5:** GTL and GC are positively correlated.

### Mediation through green creativity

In some studies, environmental and green management expertise fosters creative culture, influencing employees’ work creativity (Darvishmotevali et al., 2020). Employees who engage in green innovation approach environmental issues uniquely and creatively (Tuan, 2020). Furthermore, when GTL is spread throughout the organization, green innovations significantly impact green practices (Mittal and Dhar, 2016). In addition to the earlier discussion of the relationship between GTL, GK, and EP, green creativity is hypothesized to act as a mediator.

**H6:** GC is a measure of the association between EP and GK.

**H7:** GTL leads to GC, which describes the link between EP and GTL.

**FIGURE 1:**
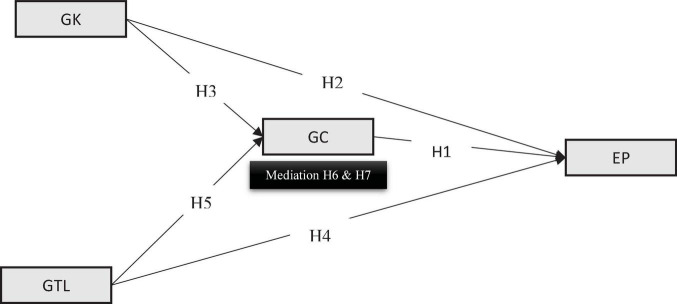
Conceptual framework.

Figure 1 presents the theoretical model derived from the relationships established above and underpinned the theories for specifying the interlinked philosophies.

## Research methods

### Sample and data collection

About 3.3 million Pakistani small medium companies exist, and their involvement to GDP is generative. In numerous sectors, roughly 78% of the labor force is engaged in non-agricultural work, with the continuing 22% establishing the commercial workforce (Shah and Syed, 2018). The Small and Medium Enterprises Development Authority (SMEDA) is an independent consultant in Pakistan that screens and delivers to SMEs a helpful support. Furthermore, SMEDA has documented various SMEs and characterized their facilities (SMEDA, 2020).

A survey was conducted in multiple cities of Pakistan, including Karachi, Lahore, Islamabad, and Quetta, to gather information from small and medium-sized businesses. Using a self-administered questionnaire, data were collected from sample fields to ensure a typical sample. Representatives who have taken a proactive role in implementing green exercises were followed for data. In order to gather the information, surveyors are used. There are as numerous outlooks on whether or not purposive sampling is simple and straightforward as there are on how difficult it is. Purposive sampling is justified by the need to better align the sample with the research goals and objectives, enhancing the validity of the findings and the study’s rigorousness and delivers authenticity in the data results. Reliability, credibility, transferability, and verifiable are the four components of this notion that have been previously reported by many scholars (Campbell et al., 2020).

Hence, the study pursued “purposive sampling” recognized as judgmental, careful, or particular sampling, is a type of non-probability method where scholars depend on judgment when selecting participants of the population to take part in survey sampling (Vehovar et al., 2016).

Cochran (1977) suggested that when the population is unknown, there is an adequate method to identify the sample from the unknown population using a following formula. Also suggested by prior scholars in the context of developing countries (Qalati et al., 2021a).


$$n=\frac{z^{2}}{4\,e^{2}},n=\mathrm{sample}\,\mathrm{size}$$

p=thepopulationratios

e=adequatesamplingerror(e=5%)

$$n=\frac{{\left(1.96\right)}^{2}}{4\,{\left(0.05\right)}^{2}}=384.16$$

Researchers explained the study’s purpose to each respondent before asking them to complete the questionnaire, as instructed by the investigators. Following their agreement, a questionnaire was filled out.

A total of 400 representatives from 84 of the chosen companies participated in the survey. The total number of valid responses examined was 400. In Table 1, 70% of respondents hold a bachelor’s degree, followed by 20% with a master’s, 5% with a doctorate, and 5% with other professional degrees. Among the respondents, 15% were female, while 85% were male. Age, 25–35, 46–45, and above 46 were 30, 50, and 20%, respectively.

**TABLE 1:** Demographics.

Variables	Frequency	Ratio
*Education*		
Bachelor	280	70
Master	80	20
Doctorate	20	5
Professional Degrees	20	5
*Gender*		
Male	340	85
Female	60	15
*Age*		
25–35	120	30
36–45	200	50
46–above	80	20
*Representatives levels*		
Executive	64	16
Frontline	60	15
Manager	120	30
Supervisor	96	24
Bottom-line	60	15
*Experience (years)*		
>15	184	46
10–14	100	25
<10	116	29
*Targeted segments*		
Manufacturing	120	30
Trading	180	45
Other mixed services	100	25

The study included enterprise representatives as respondents. We examined the responses from frontline to senior managers. There was a 16% executive ratio, followed by a 15% frontline manager ratio and a 30% middle manager ratio, holding supervisory designations at 24% and bottom-line (operational) managers at 15%. In the survey, 46% of respondents had more than 15 years of experience, 10–14 25% and less than ten 29% were from small enterprises.

Furthermore, distributed industry services, the study targeted manufacturing 30%, trading 45%, and mixed services (education, healthcare, trainings, and etc.) 25% involved for study purpose.

### Pre-testing and a questionnaire

Measurements of fundamental constructs were based on instruments from earlier surveys. Measures included a variety of components. EP as a dependent construct measured having three statements that were adjusted from Chiou et al. (2011). Two independent constructs GTL and GK were assessed by six and four questions modified and used from the work of Chen et al. (2014), and Subramanian et al. (2016), separately. GC measurement used six statements acquired and modified from Chen et al. (2014). Likert scale based on 5-ranks (one represents strongly disagree to five shows strongly agree) was used for independent and mediating constructs. Details of the items are given in Appendix. The tool’s validity was evaluated by five experts, including two human resource managers from the sample companies and three university professors having expertise in it. As a result of their advice, a few minor adjustments have been made. The questionnaire was piloted with 45 participants to ensure it was clear and feedback was collected. Changes did not need to be made to the questionnaires. Reliability analysis was also performed on the scales utilized in the study to assess their internal consistency.

We included small and medium-sized businesses in our research because they are vital to economic growth. By participating in international trade, SMEs contribute to the development of the global economy and create jobs. Small and medium-sized businesses explain around 90% of trade and employment worldwide. SMEs contribute approximately 40% of the national income in emerging economies. In order to keep up with the growth of the global workforce, approximately 600 million jobs will be required by 2030, according to World Bank estimates (World Bank, 2021). Since implementing globalization strategies, small and medium-sized companies have expanded their operations.

## Results analysis

### Descriptive data analysis

Regarding descriptive analysis, for EP, the mean value of 4.3 and the standard deviation (STD) of ±0.72 show that average respondents fell in agreement with statements with variation (3.58 to 5), respectively. The mean value for the green knowledge is 4.08, and the standard deviation is ±0.64, showing that this deviation ranges from 3.44 to 4.72. This suggests that agreed statements were observed. According to the mean value of 4.06, the respondents comprehend the average GC agreement. The standard deviation demonstrates the deviation ranging from 3.46 to 4.66. Regarding the GTL, the mean value of 4.00 shows that, on average, they went for positively agreed with the statements. Also, the standard deviation of ±0.67 shows this deviation, which ranges from 3.33 to 4.67; displayed in Table 2.

**TABLE 2:** Descriptive statistics.

Constructs	Mean	STD	CA	CR	AVE
Environmental performance	4.30	0.72	0.92	0.94	0.86
Green knowledge	4.08	0.64	0.90	0.93	0.77
Green creativity	4.06	0.60	0.84	0.88	0.56
Green transformational leadership	4.00	0.67	0.90	0.93	0.68

STD, standard deviation; AVE, average variance extracted.

### Measurement model

A measurement model evaluates convergent and discriminant validity, where convergent validity pertains to the level at which multiple items represent the same variable or concept. AVE, factor loads, and composite reliability (CR) can all be used to assess convergent validity, and all items exceeded the limit of 0.50 (Fan et al., 2021). Further, Hair et al. (2017) used 0.50 and 0.7 as cutoff values for AVE and CR, respectively. Further supporting the dependability of the measurement items was Cronbach’s alpha (CA), which indicated higher than 0.70 for all variables. A factor loading threshold of 0.6 and variance inflated factor (VIF) less than 3.3 (Qalati et al., 2022b) are considered standards. Tables 2, 3 show the output of the measurement model.

**TABLE 3:** Factor analysis.

Environmental performance	FL	Inner VIF
EP1	0.929	
EP2	0.923	
EP3	0.927	
Green knowledge		1.824
GK1	0.89	
GK2	0.871	
GK3	0.878	
GK4	0.866	
Green creativity		1.963
GC1	0.681	
GC2	0.686	
GC3	0.772	
GC4	0.831	
GC5	0.776	
GC6	0.739	
Green transformational leadership		1.248
GTL1	0.807	
GTL2	0.858	
GTL3	0.832	
GTL4	0.816	
GTL5	0.823	
GTL6	0.791	

FL, factor loading; VIF, variance inflated factor.

**FIGURE 2:**
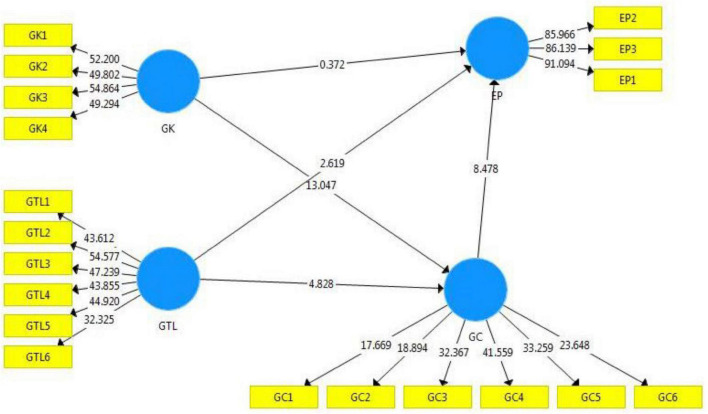
Structural model.

As per Fornell and Larcker (1981), discriminant validity is an important check for validating the data. Also, correlational numbers identify that the square root of AVE is considered higher than all other correlations within a column, as presented in Table 4. This study fulfilled. The Heterotrait-Monotrait ratio (HTMT) was also utilized to evaluate discriminant validity, which measures how well a construct or set of measures distinguishes between various constructs. The HTMT ratio was considered. Besides the frequently used Fornell-Larcker criterion, the method follows two different threshold values lower than 0.85 and 0.90, a benchmark by Henseler et al. (2015). In the study, 0.85 was used as the cutoff point for evaluating discriminant validity, and the outcome was satisfactory (Table 4). Therefore, discriminant validity and convergent validity determined which outcomes were acceptable for the measurement model (Figure 2).

**TABLE 4:** Discriminant validity.

Constructs	1	2	3	4
**Fornell and Larcker**				
EP	*0.926*			
GC	0.637	*0.749*		
GK	0.446	0.668	*0.876*	
GTL	0.377	0.437	0.359	*0.821*

**Constructs**	**EP**	**GC**	**GK**	**GTL**

**HTMT (Heterotrait-Monotrait) ratio**				
EP	–			
GC	0.725			
GK	0.491	0.766		
GTL	0.41	0.494	0.397	–

Underline value reflects the square root of the AVE.

### Structural model

The employability of Smart-PLS has been accepted widely in different fields, especially social and management sciences (Zafar et al., 2022). Hence, the researchers used this software package. It is essential to consider the coefficient of determination (R^2^), the significance of the b-path coefficient, the effect size (f^2^), and the cross-validated redundancy (Q^2^) when evaluating a structural model evaluation (Hair et al., 2017). Cohen (1992) suggests the following ranges to understand R^2^ values: low, 0.02–0.12; moderate, 0.13–0.25; and significant, 0.26 and above. A study of moderate EP found that it was explained by GTL and GC (Cohen, 2013) (R^2^ = 0.419); see Table 5. The default model achieves the square root of mean residual (SRMR = 0.05) and normed fir index (NFI ≅ 0.9) values in acceptable range; see Table 5.

**TABLE 5:** Model fit and predictive relevance.

Variables	Q^2^	f^2^	R^2^	SRMR	NFI
EP	0.623	–	0.419	0.051	0.883
GC	0.383	0.284	0.488		
GK	0.566	0.00	–		
GTL	0.521	0.02	–		

Table 6 depicts that a positive association between GC and EP (β = 0.570, *p* > 0.01, *t* = 8.58), but GK does not significantly influence RP (β = 0.022, *p* < 0.05, *t* = 0.709), which contradicts H2, but supports H1. In contrast, it was discovered that GK significantly ensured a positive relation with GC (β = 0.587, *p* > 0.01, *t* = 13.04) and that GTL had a positively significant relation with EP (β = 0.121, *p* > 0.01, *t* = 2.60), sustaining hypotheses H3 and H4. Hypothesis H5 is also supported as GTL positively influenced the GC by the analysis result of (β = 0.226, *p* > 0.01, *t* = 4.875).

**TABLE 6:** Hypotheses results.

Hypothesis	Relationship	Beta	*t*-statistics	*p*-values	Decision
H1	GC → EP	0.570	8.588	0.000	Supported
H2	GK → EP	0.022	0.373	0.709	Not supported
H3	GK → GC	0.587	13.040	0.000	Supported
H4	GTL → EP	0.121	2.606	0.009	Supported
H5	GTL → GC	0.226	4.875	0.000	Supported
**Mediation test**					
H6	GK → GC → EP	0.334	6.076	0.00	Supported
H7	GTL → GC → EP	0.129	4.607	0.00	Supported

Green creativity was also examined as a mediator between EP, GTL, and GK. Mediating impacts are significant if there is an indirect link between independent and dependent variables (Preacher and Hayes, 2008). GC was found to hold a significant mediation impact on the relationship between GK and EP (β = 0.334, *p* > 0.01, *t* = 6.07), as well as on the relationship between GTL and EP (β = 0.129, *p* > 0.01, *t* = 4.60), supporting hypotheses H6 and H7 respectively, shown in Table 6 and Figure 2. As a final step, the predictive significance of the model was evaluated using blind-folding at a distance of 7.

The model has a predictive value if Q^2^ is bigger than 0 for the specified endogenous constructs (Hair et al., 2017). For EP and GC, Q^2^ was greater than zero and greater than 0.118, indicating adequate predictive relevance (Table 5).

## Discussion

This study examines how GTL and GK affect RP and assesses how GC can mediate this effect.

Unlike early studies, the results suggest no substantial connection between understanding environmental issues and EP (Amoah and Addoah, 2021). A successful GK initiative results from information creation, knowledge transfer, and knowledge application (Wu and Haasis, 2013; Cheng, 2019). As a result, one justification for this conflicting result could be that operators have a sufficient understanding of environmental concerns but find it challenging to apply these skills. Moreover, having a green mindset does not just mean knowing what environmental issues are; it also means knowing what to do about them. In general, there is no clear link between knowledge and attitudes, and the usefulness of information depends mainly on environmental attitudes that are dependent on GK (Liefländer and Bogner, 2018). Employee behavior is influenced by social factors, such as support for the community (Safari et al., 2018). In a nutshell, higher authorities’ assistance, awareness, and concern could facilitate the environmental knowledge of managers and help them increase their performance within the environment (Majumdar and Sinha, 2019). A scarcity of GK, cognizance and care by top authorities can significantly negatively impact the EP of enterprises. To manage an enterprise’s environmental system and promote green awareness among managers and coworkers, green HRM must pay more attention to designing and delivering the proper training and development degree. Thus, a green knowledge management system is recommended.

Except green knowledge all hypotheses are supported, this study expresses that SMEs hold intention to work for social and environmental issues and their participation is highly regarded. Green creativity plays its major role for environmental productivity. Besides, between GTL and EP also proves its contribution that leadership helps to achieve environmental performance direct and through green creativity. In another way, green knowledge helps improving environmental productivity. In addition, it also partakes through green creativity to achieve environmental. Green transformational also fulfills the SMEs to attain environmental performance.

### Theoretical implications

Environmental performance has become an essential and influential topic for many scholars, as was previously supposed. Numerous studies are being conducted to identify the multiple factors that affect EP. Our research first looked into how environmental beliefs influence EP in businesses. Second, to close the information gap, this study affixes to the disciplinary knowledge by exploring how green creativity intermediates the relation between GTL and green environmental practices in SMEs. Second, the measurement strategy that examines the influence of values would add to the body of knowledge. Third, the researcher concludes that incorporating GK into practices will not necessarily improve EP. Fourth, the research confirmed the importance of GC as a mediator for enhancing ecological performance (Renwick et al., 2013). Consequently, a recent study examining the influence of GC as a mediator to strengthen or alter the association between GK and EP has suggested an important contribution to enhancing EP.

### Managerial implications

The research offers crucial recommendations for managers and leaders on fostering green practices and using them to boost EP and overtake market rivals. Consistent with our research findings, businesses should support and develop green leadership traits to apply green human resources management methods. The GC and GK division must implement green procedures to draw in, train, and keep employees who share its commitment to ethical business practices and environmentally friendly goods and services. We advise transforming small-medium enterprises’ leadership to foster an environment where individuals with green talents and drive impressions are supported and have the chance to reach their full potential to stay up-to-date and competitive.

### Limitations and prospective directions

First, it is guided that future studies look at other cities of Pakistan encompassing vast geographic areas, including growing divisions since the data was gathered from SMEs operating in major cities in Pakistan. Second, the EP scale is usually employed to gauge the impact of environmental values using the practice of green management activities. To further evaluate the hypothesis’s findings, it is advised that researchers examine the ecological importance of construction at the employee level using the Best/Worst Questionnaire. Third, this study employed GC as a mediator; it is advised that future research can think through pro-environmental activities or environmental knowledge as moderators.

## Data availability statement

The raw data supporting the conclusions of this article will be made available by the authors, without undue reservation.

## Ethics statement

The studies involving human participants were reviewed and approved by Sukkur IBA University. The patients/participants provided their written informed consent to participate in this study.

## Author contributions

All authors listed have made a substantial, direct, and intellectual contribution to the work, and approved it for publication.

## Appendix

**Table:** Measurement scale items.

S.No	EP	1	2	3	4	5
1	“My organization is focused to reduce hazardous waste”					
2	“My organization is focused to consume fewer natural resources (such as water and gas)”					
3	“My organization is interested to comply with environmental regulation”					
	**GK**					
1	“I know green products and services can reduce negative impact on the environment”					
2	“I know how to implement green practices and strategies”					
3	“I collect information about environmental issues”					
4	“I appreciate green marketing practices adopted by business entities”					
	**GC**					
1	“Employees suggest new ways to adopt green practices”					
2	“The organization adopt new ways to increase environmental compliance”					
3	“Sometimes we discuss to come with new green service ideas or market offering”					
4	“Adequate planning is taken to implement new green ideas”					
5	“My organization is receptive enough to adopt new green ideas to solve environmental problems”					
6	“My company actively participates in green innovation and technological adoption”					
	**GTL**					
1	“I motivate the employees to come with innovative green ideas”					
2	“I work with my employees to obtain environmental goals”					
3	“I inspire the employees to comply with environmental compliance”					
4	“I provide clear vision regarding environments to the employees”					
5	“I stimulate the employees to think about the environmental degradation”					
6	“I come up with answers of environmental problems which frequently raise”					
